# Learnings From a Mixed Model Communication Skills Training for International Medical Graduates

**DOI:** 10.1192/bjo.2025.10259

**Published:** 2025-06-20

**Authors:** Harleen Kaur Birgi, Akshith Shetty, Patrick Morris, Lisa Harrison, Rahul Bhattacharya

**Affiliations:** 1 East london NHS Foundation Trust, London, United Kingdom; 2 Talking Allowed, London, United Kingdom

## Abstract

**Aims:** International Medical Graduates (IMGs) make up more than half of new recruits in the NHS and recent data shows that there is an increased likelihood of IMGs being referred to the General Medical Council (GMC) for fitness to practice investigations. Analysis of these complaints highlighted communication skills as an area of concern. We wanted to support relatively new IMGs, who started practicing in the country within the last 2 years, by enhancing their communication skills.

**Methods:** To deliver this initiative, medical education department, trust IMG tutor and two higher trainees (with recent lived experience as an IMG in the NHS) partnered with Talking Allowed (communication skills training company for healthcare professionals). 12 IMGs registered and were offered 5 half day online and 1 day face to face training using a mixed model of activities related to all aspects of communication – verbal, non-verbal and written. We ran role plays on: *‘Managing an upset relative’, ‘Breaking Bad News’, ‘Misunderstandings and Colloquialism’, ‘Dealing with disagreement/ conflict within the team’* and *‘Challenges to professional boundaries’.*

During role plays, prior to candidates’ attempt, a ‘how not to do it’ version was acted out by the higher trainees followed by the enrolled participants going next. Each role play was further followed by reflective feedback sessions facilitated by PM, LH & RB to identify and reflect on positive as well as areas for improvement. Through the course one session focused on written communication, with homework and best practice guidance provided prior to the session. At the last session, there was a didactic presentation and discussion on acculturation with opportunities to reflect. Feedback was gathered at every session from the attending IMGs. 7–8 trainees engaged through the process, 2 dropouts and 2 non-engagements.

**Results:** 97.1% (n=35) rated the sessions as ‘highly engaging’ (on a scale of 1 to 4), 100% rated the sessions as ‘highly relevant’ (n=36); 91.4% (n=35) rated the course as ‘highly useful’. Compared with at the start of the course, subjective confidence in communication of participants improved by 2 points in a scale of 0 to 10. Initial score 6.8 and final score 8.8 (signifying 20% increase).

**Conclusion:** Focused communication skills training involving a mixed method of role play using simulation principles, complemented by general reflective exercises on communication especially in a cultural and emotional context as well as some reflection on the personal experience and the known intelligence on immigration and acculturation can be an invaluable tool for IMGs.

